# Validating Rat Model of Empathy for Pain: Effects of Pain Expressions in Social Partners

**DOI:** 10.3389/fnbeh.2018.00242

**Published:** 2018-10-17

**Authors:** Chun-Li Li, Yang Yu, Ting He, Rui-Rui Wang, Kai-Wen Geng, Rui Du, Wen-Jun Luo, Na Wei, Xiao-Liang Wang, Yang Wang, Yan Yang, Yao-Qing Yu, Jun Chen

**Affiliations:** ^1^Institute for Biomedical Sciences of Pain, Tangdu Hospital, The Fourth Military Medical University, Xi'an, China; ^2^Key Laboratory of Brain Stress and Behavior, PLA, Xi'an, China

**Keywords:** empathy, pain, pain hypersensitivity, pain model, rat

## Abstract

Pain can be socially transferred between familiar rats due to empathic responses. To validate rat model of empathy for pain, effects of pain expressions in a cagemate demonstrator (CD) in pain on empathic pain responses in a naïve cagemate observer (CO) after 30 min priming dyadic social interactions (PDSI) were evaluated. The CD rats were prepared with four pain models: bee venom (BV), formalin, complete Freund's adjuvant (CFA), and spared nerve injury (SNI). Both BV and formalin tests are characterized by displayable and eye-identifiable spontaneous pain-related behaviors (SPRB) immediately after treatment, while CFA and SNI models are characterized by delayed occurrence of evoked pain hypersensitivity but with less eye-identifiable SPRB. After 30 min PDSI with a CD immediately after BV and formalin, respectively, the empathic mechanical pain hypersensitivity (EMPH) could be identified at both hind paws in CO rats. The BV—or formalin-induced EMPH in CO rats lasted for 4–5 h until full recovery. However, EMPH failed to develop in CO after socially interacting with a CD immediately after CFA, or 2 h after BV when SPRB completely disappeared. The CO's EMPH was partially relieved when socially interacting with an analgecized CD whose SPRB had been significantly suppressed. Moreover, repeated exposures to a CD in pain could enhance EMPH in CO. Finally, social transfer of pain hypersensitivity was also identified in CO who was being co-housed in pairs with a conspecific treated with CFA or SNI. The results suggest that development of EMPH in CO rats would be determined not only by extent of familiarity but also by visually identifiable pain expressions in the social partners during short period of PDSI. However, the visually unidentifiable pain can also be transferred to naïve cagemate when being co-housed in pairs with a distressed conspecific. In summary, the vicariously social contagion of pain between familiar rats is dependent upon not only expressions of pain in social partners but also the time that dyads spent in social communications. The rat model of empathy for pain is a highly stable, reproducible and valid model for studying the neural mechanisms of empathy in lower animals.

## Introduction

Pain can be socially transferred between familiar conspecifics in rodents due to empathic responses (Martin et al., 2014; Mogil, 2015; Chen, 2018). From the point of evolutionary view, empathy can be defined as an evolutionary behavior of social animals and humans associated with prosocial reciprocity, altruism, and morality by the ability and capacity to feel, recognize, understand, and share the emotional states of others (Preston and de Waal, 2002; de Waal, 2008; Bernhardt and Singer, 2012; Decety et al., 2016; de Waal and Preston, 2017; Chen, 2018). In humans, both directly felt pain and empathy for pain has been demonstrated to be processed by a common core neural network mainly involving the anterior cingulate cortex (ACC) and anterior insular cortex (Rainville et al., 1997; Singer et al., 2004; Lamm et al., 2011). Empathy for pain (or fear) has been demonstrated to be able to modulate the feeling of pain and pain sensitivity, resulting in empathic pain hypersensitivity (hyperalgesia or allodynia) in humans (Goubert et al., 2005; Williams and Rhudy, 2007; Loggia et al., 2008; Villemure and Bushnell, 2009; Godinho et al., 2012).

Recently, empathy for pain or distress has been proposed to be mediated by common neural and neuroendocrine processes in both humans and animals (for reviews see Panksepp and Lahvis, 2011; Gonzalez-Liencres et al., 2013; Panksepp and Panksepp, 2013; Martin et al., 2014; Mogil, 2015; Keum and Shin, 2016; Meyza et al., 2016; Sivaselvachandran et al., 2016; Lü et al., 2017; Chen, 2018). In rodents, empathy for pain has been reported to consistently exist in both mice (Langford et al., 2006; Martin et al., 2015) and rats (Li et al., 2014; Lü et al., 2017; Lu et al., 2018) following social interactions between dyadic familiar (cagemate) rats but not stranger (non-cagemate) ones for which one animal is in pain (for reviews see Martin et al., 2014; Mogil, 2015; Chen, 2018). It is of special interest to note that familiar relationship among conspecifics is essential to induction of empathic responses to other's pain in rodents following habituation in the same cage for more than 2 weeks (Martin et al., 2014; Mogil, 2015; Chen, 2018). We have found that after 30 min priming social interactions with a familiar rat in pain evoked by unilateral subcutaneous (s.c.) injection of bee venom (BV), the naïve observer rat shows empathic mechanical (but not thermal) pain hypersensitivity (EMPH) (Li et al., 2014; Lü et al., 2017; Lu et al., 2018). The familiarity-based EMPH identified in naïve observer rats has been shown to be mediated by top-down facilitation from the medial prefrontal cortex (mPFC) (Li et al., 2014). Moreover, the locus coeruleus (LC)-norepinephrine (NE) system which is probably driven by activation of the mPFC has also been demonstrated to be involved in mediation of the EMPH through up-regulation of ATP P2X3 receptor subunits in the dorsal root ganglia (DRG) by increased circulating NE released from the sympatho-adrenomedullary (SAM) axis (Lü et al., 2017). Unlike the familiar social partners, however, it has been further shown that the stranger social partners (non-cagemate observers) are not likely to develop empathy for pain due to social stress driven by increased circulating corticosterone released from the hypothalamo-pituitary-adrenocortical (HPA) axis (Martin et al., 2015; Lü et al., 2017), another neuroendocrine system that is associated with social stress to various direct stressors (pain, fear, and catastrophe) (Chrousos, 2009; Ulrich-Lai and Herman, 2009). Therefore, development of empathy for pain in naïve observers is likely to be determined by the extent of familiarity between the social partners. However, besides the roles of familiarity in production of empathy for pain, the roles of other factors such as the extent of pain expressions in the social demonstrators also require to be further examined.

Thus, in the present study, the rat model of empathy for pain was further validated by assessment of the effects of pain expressions in the familiar partners (demonstrators) on the development of the EMPH in the naïve observers after priming dyadic social interactions (PDSI). Because it has been demonstrated that the visual information is essentially important for induction of empathy for pain (Langford et al., 2006) and social contagion of itch (Yu et al., 2017) in mice, we provided with different individual demonstrator prepared by different pain models, respectively, as different visual cues for the naïve observer rats in the setting of dyadic social interactions. In one set of experiments, social transfer of pain was studied after 30 min dyadic social interactions between a naïve observer and a familiar conspecific (demonstrator) who had visually identifiable spontaneous pain-related behaviors (SPRBs) or not. In another set of experiments, social transfer of pain was also studied when a naïve rat was being co-housed for 2 weeks with a distressed conspecific who had evoked pain hypersensitivity but with less or no visually identifiable SPRBs. For both sets of experiments, pain behaviors were recorded and quantified in both observer and demonstrator subjects.

## Materials and methods

### Animals

Only male Sprague-Dawley rats (180–250 g, 8–10 weeks old) were used in the present study. After arrival at the SPF animal facility at our lab, the animals purchased from the Laboratory Animal Center of the Fourth Military Medical University (FMMU), Xi'an, Shaanxi Province, P.R. China, were housed under standard conditions (12 h dark/light cycles, temperature 22–26°C, air humidity 40–60%) in a group of 6 rats with free access to food and water. The number of animals used and their suffering were minimized as possible as we could. In the present study, social partners were referred to as familiar cagemates who were co-housed in the same cage for more than 2 weeks before initiation of the experiment, and were not necessarily siblings. For each set of experiment, a new cohort of rats was used. No animals were used more than one test. The experimenters and data-processing persons were blind to the treatment of rats.

### Experimental design

**Figure 1:**
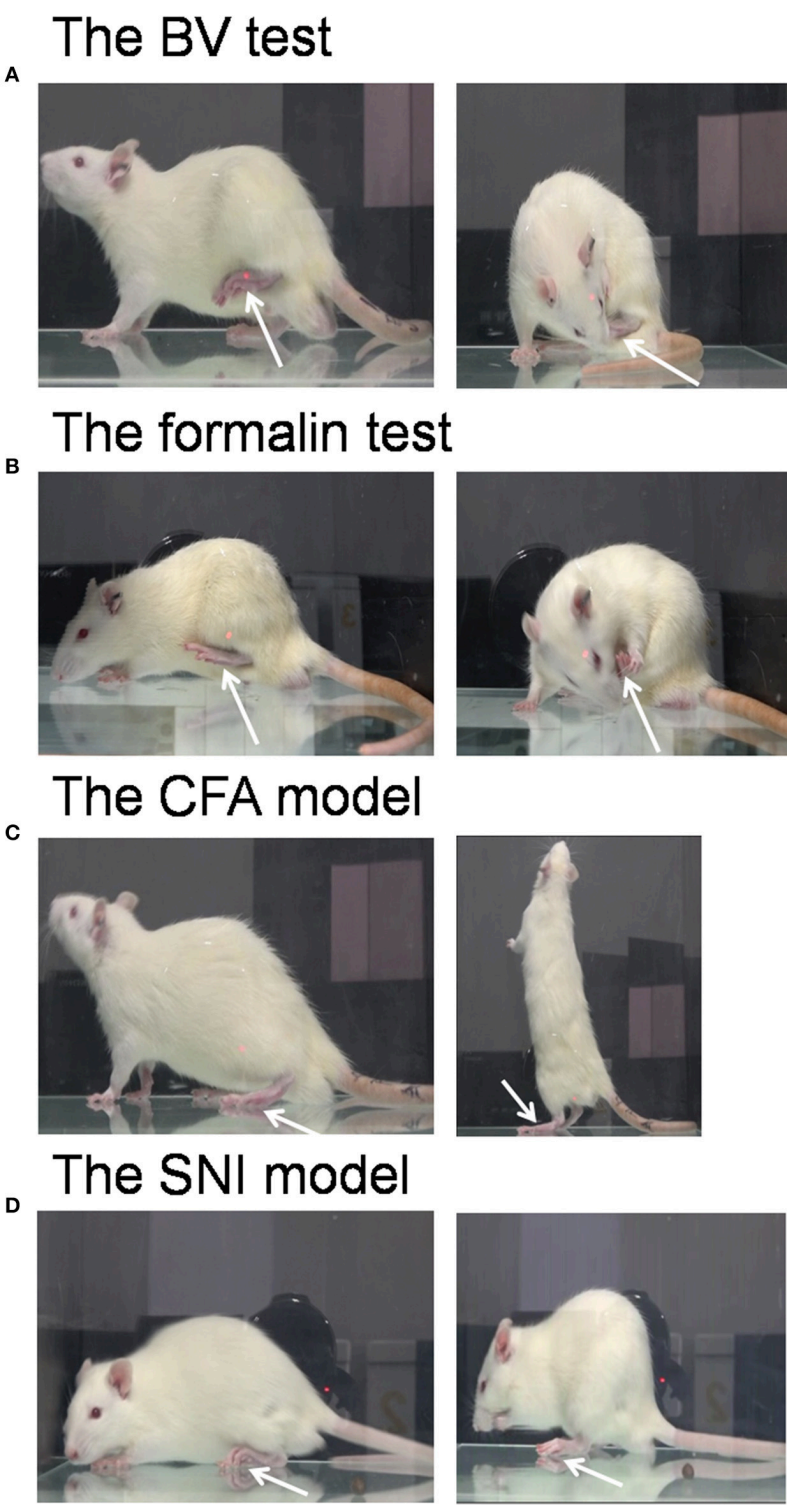
Pain models used in the demonstrator rats. The bee venom (BV) test **(A)** and the formalin test **(B)** are two models of acute inflammatory pain in which the animals have immediate long-lasting displayable (eye-identifiable) spontaneous pain-related behaviors including paw flinches and paw lifting and licking in the injured side of the hind paws following subcutaneous injection of BV or formalin solution. The complete Freund's adjuvant (CFA) model and the spared nerve injury (SNI) model are two models of chronic pain in which the animals have less spontaneous displayable pain-related behaviors but with delayed occurrence of pain hypersensitivity following the two treatments. Unlike the BV **(A)** and formalin **(B)** test, rats with CFA **(C)**, and SNI **(D)** can normally bear the body weight through the injured hind paw, and move or stand freely. The arrow indicates the injured hind paw of the demonstrator. The images for **(A–C)** were captured randomly during the first 5 min dyadic social interaction after different treatment, while that for D was taken 14 days after SNI surgery.

After being co-housed for at least 2 weeks, dyads of cagemates were randomly assigned to the following experiments. (1) The effects of displayable (eye-identifiable) and non-displayable (less eye-identifiable) pain in the demonstrators on the empathic pain responses in the naïve observers were assessed after dyadic social interactions. In this set of experiment, the BV test (Figure 1A, *n* = 8) and the formalin test (Figure 1B, *n* = 8) were selected as displayable pain models because in the initial session of 30 min observation the demonstrator rats display immediate long-lasting SPRB s with paw flinches and paw licking and lifting in the injured side of the hind paws following s.c. injection of BV or formalin solution (Figures 1A,B), while the complete Freund's adjuvant (CFA) model (Figure 1C, *n* = 8) and the late phase of BV test (**Figure 4**, *n* = 10) were selected as non-displayable pain models because the demonstrator animals display less SPRB s but with delayed occurrence of evoked mechanical pain hypersensitivity (Video for the pain expressions in a rat with BV, formalin or CFA see Supplemental Video 1). (2) The effects of analgecized demonstrators on the empathic pain responses in the naïve observers were assessed after dyadic social interactions. Lidocaine (Lido) was used as local analgesic to block the BV-induced pain-related behaviors in the demonstrators (*n* = 8) (**Figure 5A**). (3) The effects of frequent exposures to a familiar demonstrator in pain on the empathic pain responses in naïve observer were examined. In this set of experiment, a naïve observer (*n* = 10) allowed to expose to a familiar demonstrator in pain caused by s.c. BV injection. For one trial test, a naïve cagemate observer (CO) was exposed to a cagemate demonstrator (CD) only for one trial (*n* = 10 pairs); while for the three-trials test, a naïve CO was exposed to three different CDs in successive three trials with 5 min inter-trial interval (*n* = 10 for CO and *n* = 30 for CD). From a group of 6 rats co-housed in one cage, one rat was randomly served as a naïve CO, while other cagemates served as a demonstrator for each trial observation in the testing box. Each trial had 30 min in duration starting from the time when a CD was placed in the testing box immediately after s.c. BV injection for dyadic social interactions with the CO (**Figure 6A**). The paw withdrawal mechanical threshold (PWMT) was measured for 5 h (with 1 h interval) immediately after trial 1 (indicated by a downward red arrow in **Figure 6B**), while for the three-trials test, the PWMT was measured only once after trial 1 and trial 2, respectively, and but measured for 5 h after trial 3 (indicated by upward blue arrows in **Figure 6B**). (4) Social transfer of non-displayable mechanical pain hypersensitivity between an ill and a naïve cagemate was examined when they were being co-housed in pairs for 2 weeks. The ill rats were treated with s.c. injection of CFA (Figure 1C, *n* = 10) or spared nerve injury (SNI, Figure 1D and Supplemental Video 1, *n* = 9). Cagemate control (CC) was a set of experiment allowing two pain-free cagemate rats to socially interact for 30 min or to be co-housed for 2 weeks.

### Priming dyadic social interaction

**Figure 2:**
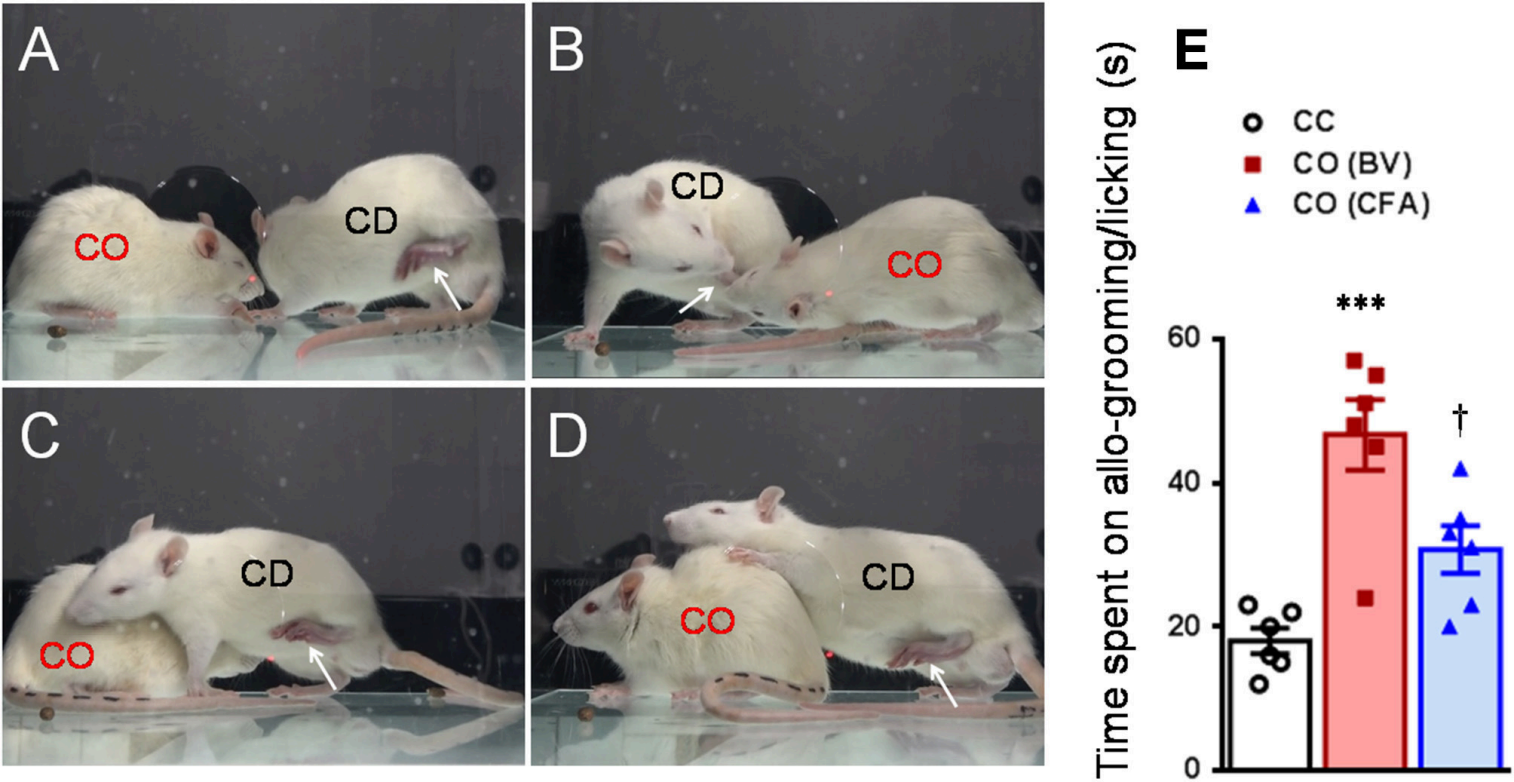
Consolation behaviors of a naïve observer toward a familiar demonstrator without any treatment or treated with bee venom (BV) and complete Freund's adjuvant (CFA) during 30 min priming dyadic social interaction. **(A–D)** showing consolation through social approach **(A)**, allo-licking **(B)**, and body-supporting **(C,D)** behaviors of a naïve cagemate observer (CO) toward the cagemate demonstrator (CD) in pain caused by subcutaneous injection of BV. **(E)** Showing quantitative comparisons of the time CO rats spent on allo-grooming/allo-licking behaviors toward a CD without any treatment (CC) and treated with BV (CO [BV]) or CFA (CO [CFA]) during 30 min priming dyadic social interaction. The arrow indicates the injured hind paw of the demonstrator. ^***^P < 0.001 CO (BV) vs. CC; ^†^*P* < 0.05 CO (CFA) vs. CO (BV), *n* = 6 animals for each group.

After being acclimatized to the testing environment, one of the dyads was removed and treated with BV, formalin or CFA. As previously and above described (Li et al., 2014; Lü et al., 2017; Lu et al., 2018), immediately after s.c. injection of BV or formalin or CFA, the CD was reunited with the CO in a plastic testing box (30 × 30 × 30 cm) for 30 min social interaction (Figures 2, 6A). The number of paw flinches and the time CD rats spent on paw licking were counted by raters blind to the treatments. In some cases, video camera (FDR-AX40, SONY, Japan) was also used to record the behaviors of both the observer and the demonstrator during 30 min dyadic social interactions immediately after reunion of the dyads (Figure 2). Off-line analysis was then conducted to measure the time CO rats spent on allo-grooming and allo-licking behaviors that have been proved to be empathy-based consolation toward a distressed partner (Burkett et al., 2016; Lu et al., 2018). Allo-grooming was defined as head contact with the body or head of the stressed individual (Burkett et al., 2016), while allo-licking was defined as head contact with the injured paw pad of the stressed individual, accompanied by a rhythmic head movement. Grooming directed toward the rear (genitals, anogenital region, or tail) was excluded.

### Pain models used in the demonstrator rats

The BV test was produced by s.c. injection of BV solution (whole venom 0.2 mg in 50 μL in physiological saline, purchased from Sigma, St. Louis, MO USA) into the left hind paw of the demonstrator rats (Chen et al., 1998, 1999; Chen and Chen, 2000; for review see Chen and Lariviere, 2010). It is characterized by two distinct phases of nociceptive responses: an immediate early phase consisting of an eye-identifiable or displayable long-term persistent SPRBs displaying as paw flinching reflex and paw licking and lifting behaviors at the injection side for about 1 h which would be followed by a late phase with evoked primary pain hypersensitivity to mechanical and thermal stimuli (since 2 h after BV and last about 72–96 h) but with less eye-identifiable paw flinches and licking behaviors (Figure 1A and Supplemental Video 1, for details see Chen et al., 1999; Chen and Chen, 2000; Chen and Lariviere, 2010). The rats with BV injection limped and could not bear the body weight through the injured paw and kept paw lifting during the observation period. The BV-induced paw flinches and the time the animal spent on licking the injured paw in the early phase can be counted for quantitative analysis. Video for the pain expressions in a rat with BV was shown in Supplemental Video 1. The late phase of the BV test can be quantitatively assessed by measuring the PWMT (for details see below).

**Figure 3:**
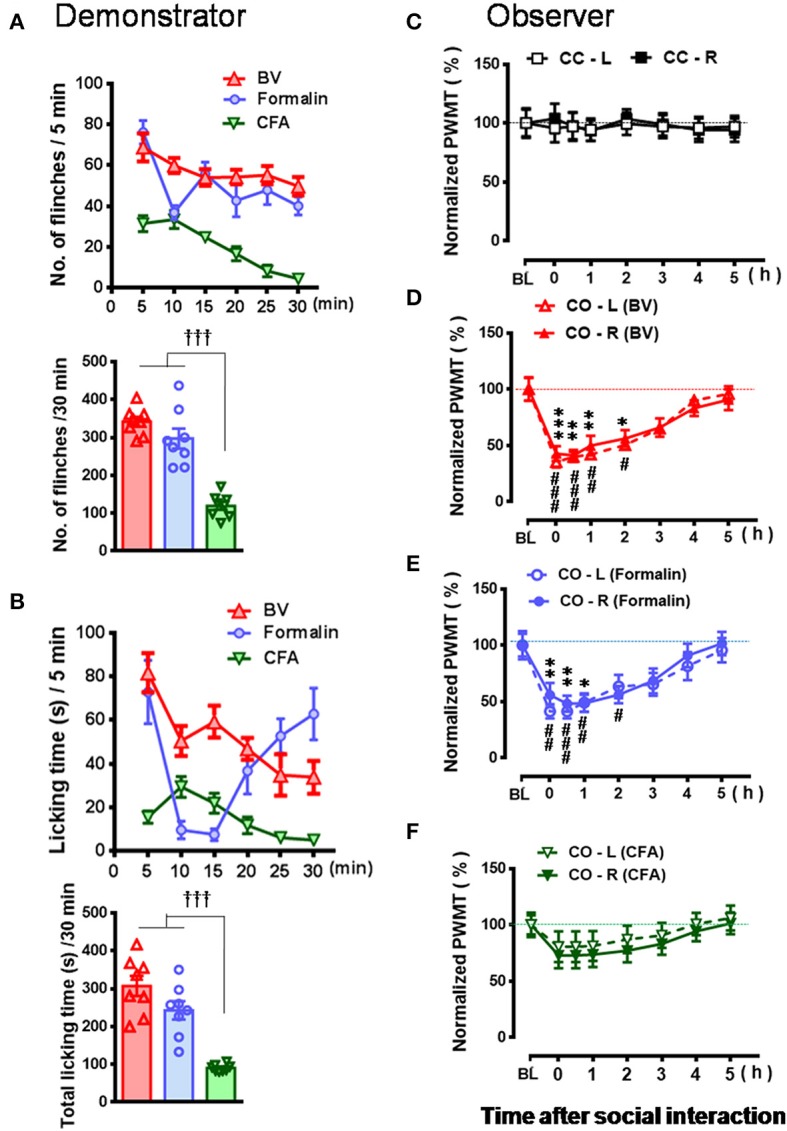
Comparative observations of the effects of three inflammatory pain models used in the familiar demonstrators on the empathic pain responses in the naïve observers. **(A,B)** showing the mean time courses and total number or time of paw flinches or paw licking behaviors that the demonstrator rats displayed during 30 min dyadic social interaction immediately after subcutaneous injection of three chemical irritants into the plantar surface of one hind paw, respectively. **(C–F)** showing the time courses of changes in paw withdrawal mechanical threshold (PWMT) in the naïve cagemate control (CC) or the naïve cagemate observer (CO) after 30 min dyadic social interaction with a familiar demonstrator without any treatment (**C**, *n* = 10) or a familiar demonstrator in pain induced by BV (**D**, *n* = 8), formalin (**E**, *n* = 8), and CFA (**F**, *n* = 10), respectively. BL, baseline; BV, bee venom; CFA, complete Freund's adjuvant; CC-L/CC-R and CO-L/CO-R, left or right hind limb of CC and CO rats. ^†††^*P* < 0.001, CFA vs. BV or formalin in **(A,B)** (One way ANOVA with Turkey's correction for multiple comparisons); ^*^P < 0.05, ^**^P < 0.01, ^***^P < 0.001 CO-L vs. BL; #*P* < 0.05, ##*P* < 0.01, ###*P* < 0.001, CO-R vs. BL (one way repeat measures ANOVA with Greenhouse-Geisser Correction). Data are expressed as mean ± SEM.

The formalin test is another displayable pain model which was produced by s.c. injection of formalin solution (2.5% paraformaldehyde in 100 μL physiological saline) into the left hind paw of the demonstrator rats (Chen et al., 1999). It is also characterized by an eye-identifiable SPRBs displaying as paw flinching reflex and paw licking and lifting behaviors at the injection side for about 1 h. However, unlike the BV test (Figures 3A,B), the formalin test has a silent period (11–20 min) between the early (0–10 min) and the late (21–60 min) phases of nociceptive responses (Figures 3A,B). It has been demonstrated that there is no primary pain hypersensitivity to either thermal or mechanical stimuli after formalin injection (Chen et al., 1998, 1999). The rats with formalin injection limped as well and could not bear the body weight through the injured paw either. Video for the pain expressions in a rat with formalin was also shown in Supplemental Video 1. The quantitative method for displayable pain-related behaviors was similar to what we did for the BV test (see above).

The CFA model was established by s.c. injection of 100 μL volume of CFA solution (1:1 dissolved in physiological saline, purchased from Sigma, St. Louis, MO, USA) into the left hind paw of the demonstrator rats (Yu et al., 2011). The CFA model is characterized by a delayed occurrence of mechanical (and thermal) pain hypersensitivity at the injured side but with less eye-identifiable paw flinches and paw licking or lifting behaviors at the early 1 h phase immediately after injection although paw edema was eye-identifiable (Figures 1C, **7A**). The rats with CFA injection can bear the body weight through the injured paw and move and stand freely with less eye-identifiable limps (Figure 1C and Supplemental Video 1).

The SNI model is a neuropathic pain model prepared according to a previous description (Decosterd and Woolf, 2000). The SNI model is comprised of an axotomy and ligation of the tibial and common peroneal nerves leaving the sural nerve intact. In brief, under anesthesia with sodium pentobarbital (40 mg/kg, i.p.), the left sciatic nerve and its three terminal branches were exposed. Then the left tibial and common peroneal nerves were tightly ligated with 5.0 silk thread and sectioned distally to the ligation, removing about 2 mm of the distal nerve stump. During the whole surgery, great care was taken to avoid stretching of the intact sural nerve. In the end, the muscle and skin were closed in layers. The rats with SNI had deformed paw at the injured side and developed bilateral mechanical pain hypersensitivity gradually since day 2 and it reached plateau since day 6 after surgery (Figures 1D, 7B). There were less eye-identifiable paw flinches and paw licking and lifting behaviors in rats with SNI (Supplemental Video 1).

### Quantitative measurement of PWMT

As for the measurement of mechanical sensitivity, a plastic box (20 × 20 × 25 cm) with a metal mesh floor was employed for the measurement of PWMT, von Frey monofilaments (with bending forces of 7.84, 19.6, 39.2, 58.8, 78.4, 98.0, 117.6, 137.2, 156.8, 176.4, 196.0, 245.0, 294.0, 441.0, and 588.0 mN) were applied perpendicularly to the bilateral hind paws of rats with 10 s intervals and 10 repeats on each side. The bending force able to elicit 50% paw withdraw reflexes was considered as the threshold (For details see Chen et al., 1999). The baseline value of PWMT (PWMT_baseline_) in the CD rats was measured prior to any pain-causing treatments, while PWMT_baseline_ in the CO rats was measured prior to social interaction with a CD in pain. The PWMT_baseline_ value of each animal was also normalized by a formula: [PWMT_baseline_/Averaged PWMT_baseline_ × 100%].

### Statistical analysis

All data were expressed as mean ± SEM. The data for PWMT were normalized by a formula: [PWMT_posttreatment_/PWMT_baseline_ × 100%]. One way analysis of variance (ANOVA) with Turkey's correction was used to conduct multiple comparisons among different pain models. One way repeat measures ANOVA was used to compare PWMT_baseline_ and PWMT_posttreatment_ at various time points after social interaction in the CO rats, if the data did not meet Mauchly's test of sphericity, Greenhouse-Geisser correction was performed. Mann-Whitney U-test was used to compare two independent samples of time course observations. *T*-test was also used to analyze means difference between two groups. Fisher's two-tailed test was used for analysis of contingency data. Only if the data were conformed to a normal distribution they were subjected to parametric analysis, otherwise they would be subjected to non-parametric analysis. All statistical analyses were performed using GraphPad Prism software version 6.01 and SPSS 17.0. *P* < 0.05 was considered to be statistically significant. The artwork was created by GraphPad Prism 6.01 (GraphPad Software Inc. California, U.S.A.).

## Results

### Effects of social partner's pain expression on the induction of EMPH in the naïve observer rat

As shown in Figures 2A–D and Supplemental Video 2, a naïve CO rat showed sharing and care by consolation through social approach, allo-grooming/allo-licking, and body-supporting toward the CD rat with eye-identifiable or displayable SPRBs. Specifically, the time that CO rats spent on allo-grooming and allo-licking toward the dyadic CD during 30 min social interaction was significantly longer than CC rats [Figure 2E, CO [BV] vs. CC, *F*_(2, 15)_ = 16.40, *P* < 0.0001, *n* = 6 for each group, One way ANOVA with Turkey's correction]. However, the CO rat showed less consolation toward the CD rat with CFA injection which showed less eye-identifiable SPRBs during 30 min social interaction [Figure 2E, CO [CFA] vs. CC, *F*_(2, 15)_ = 16.40, *P* > 0.05, *n* = 6 for each group, One way ANOVA with Turkey's correction]. Namely, CO rats paired with CD showing distinctly observable pain-related behaviors had more consolation behaviors than those paired with less distinguishable pain-related behaviors [Figure 2E, CO [BV] vs. CO [CFA], *F*_(2, 15)_ = 16.40, *P* < 0.05, *n* = 6 for each group, One way ANOVA with Turkey's correction] [data for CO [formalin] was not available]. Pain-related behaviors in CD rats were significantly different between the BV/formalin and the CFA treatments (Figures 3A,B, Flinch: *F*_(2, 21)_ = 42.23, *P* < 0.001, CFA vs. BV or Formalin; Licking: *F*_(2, 21)_ = 28.71, *P* < 0.001, CFA vs. BV or Formalin, one way ANOVA with Turkey's correction for multiple comparisons).

Similar to our previous results (Li et al., 2014; Lü et al., 2017), the EMPH displaying as distinct reductions in the PWMT were identified on the bilateral hind paws in CO rats after 30 min dyadic social interactions with the BV- and formalin-treated CD rats (Figures 3D,E, vs. PWMT_baseline_, ^*^/#*P* < 0.05, ^**^/##*P* < 0.01, ^***^/###*P* < 0.001, *n* = 8 for both CO [BV] and CO [Formalin], one way repeat measures ANOVA with Greenhouse-Geisser Correction). The EMPH in the CO rats could be identified immediately after social interactions and lasted for at least 4–5 h until full recovery (Figures 3D,E). However, the CO rats which had socially interacted with the CFA-treated CD rats showed less change in the PWMT on the bilateral hind paws (Figure 3F, vs. PWMT_baseline_, *P* > 0.05, *n* = 10, one way repeat measures ANOVA with Greenhouse-Geisser Correction). No change was identified in the CC rats (Figure 3C).

**Figure 4:**
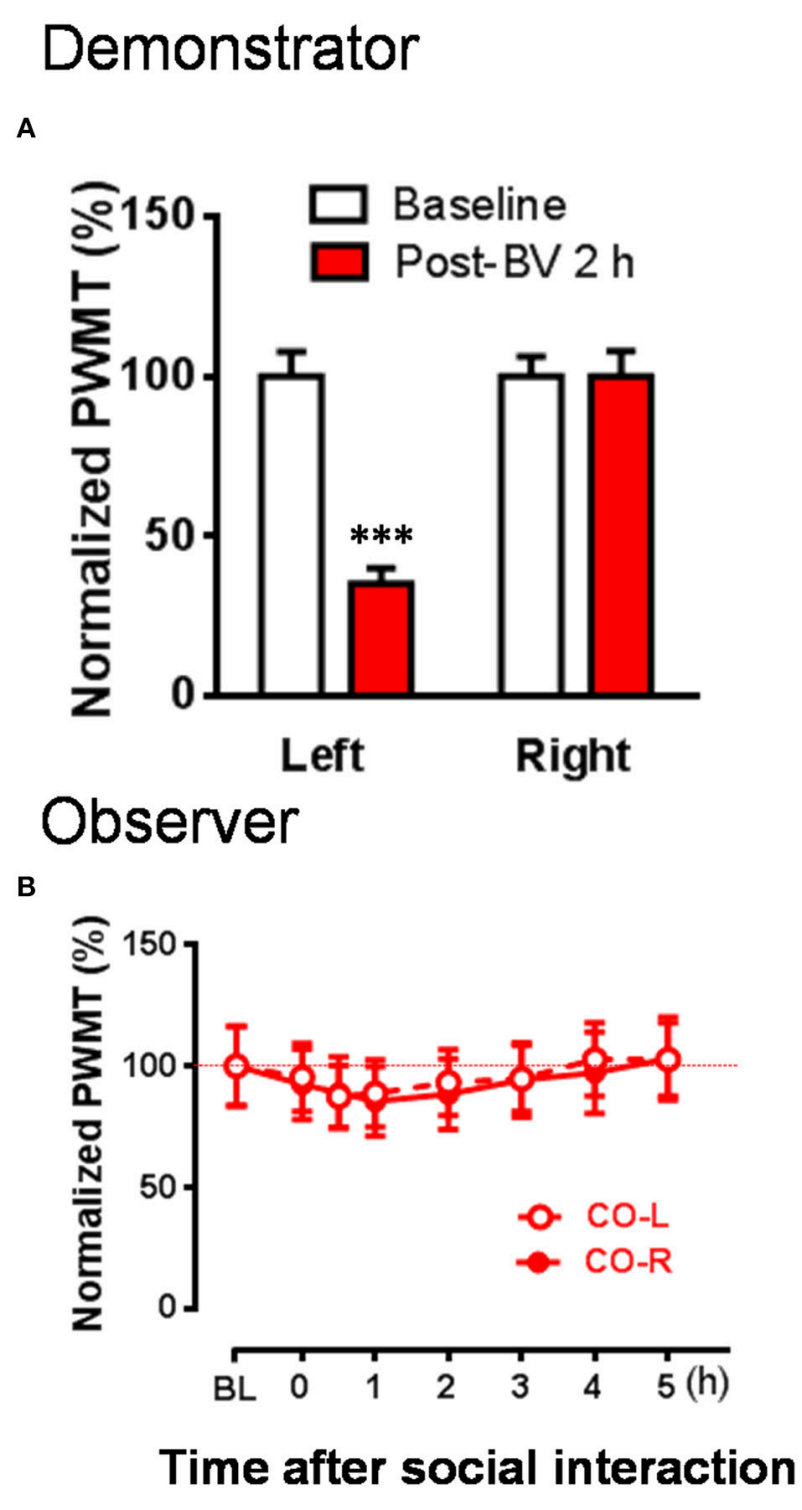
The changes in PWMT in naïve observer rats after 30 min dyadic social interaction with an familiar demonstrator whose displayable spontaneous pain-related behaviors completely disappeared while mechanical pain hypersensitivity remained 2 h after BV injection. **(A)** Showing % changes in PWMT on the injured hind paw (Left) and non-injured hind paw (Right) of demonstrator rats (*n* = 10) 2 h after BV injection. **(B)** Showing time courses of % changes in PWMT on left (CO-L) and right (CO-R) hind paws of naïve observer rats (*n* = 10) after 30 min dyadic social interaction with a familiar demonstrator receiving BV injection for 2 h. BL, baseline; BV, bee venom; PWMT, paw withdrawal mechanical threshold. ^***^ *P* < 0.001, vs. BL (Paired samples *t*-test). Data are expressed as mean ± SEM.

To re-confirm whether the CD rats' eye-identifiable SPRBs play a role in the induction of the EMPH, CO rats allowed to interact socially with another CD rats whose paw flinches and paw lifting/licking behaviors disappeared completely but the evoked pain hypersensitivity remained intact in the injection side 2 h after BV injection (Figure 4A, *P* < 0.001, vs. PWMT_Baseline_, paired samples *t*-test). No EMPH was identified at all under such priming condition (Figure 4B, vs. PWMT_baseline_, *P* > 0.05, *n* = 10, One way repeat measures ANOVA with Greenhouse-Geisser Correction).

To exclude the individual difference between animals, we counted the number of CO rats which developed the EMPH. The results showed that 80% of the CO rats (8/10) developed the EMPH (40% minimal reduction in the PWMT as standard) after socially interacting with the BV- and formalin-treated CD rats, however, only 40% (4/10) or 20% (2/10) of the CO rats showed distinct reduction in the PWMT after priming social interactions with the CD rats treated with CFA or 2 h after BV.[2 sided *P* = 0.17, CO [BV/Formalin] vs. CO [CFA]; 2 sided *P* = 0.024, CO [BV] vs. CO [Post-BV 2 h], Fisher's two-tailed test].

### Pre-blockade of social partner's pain relieved EMPH in the naïve observer rat

**Figure 5:**
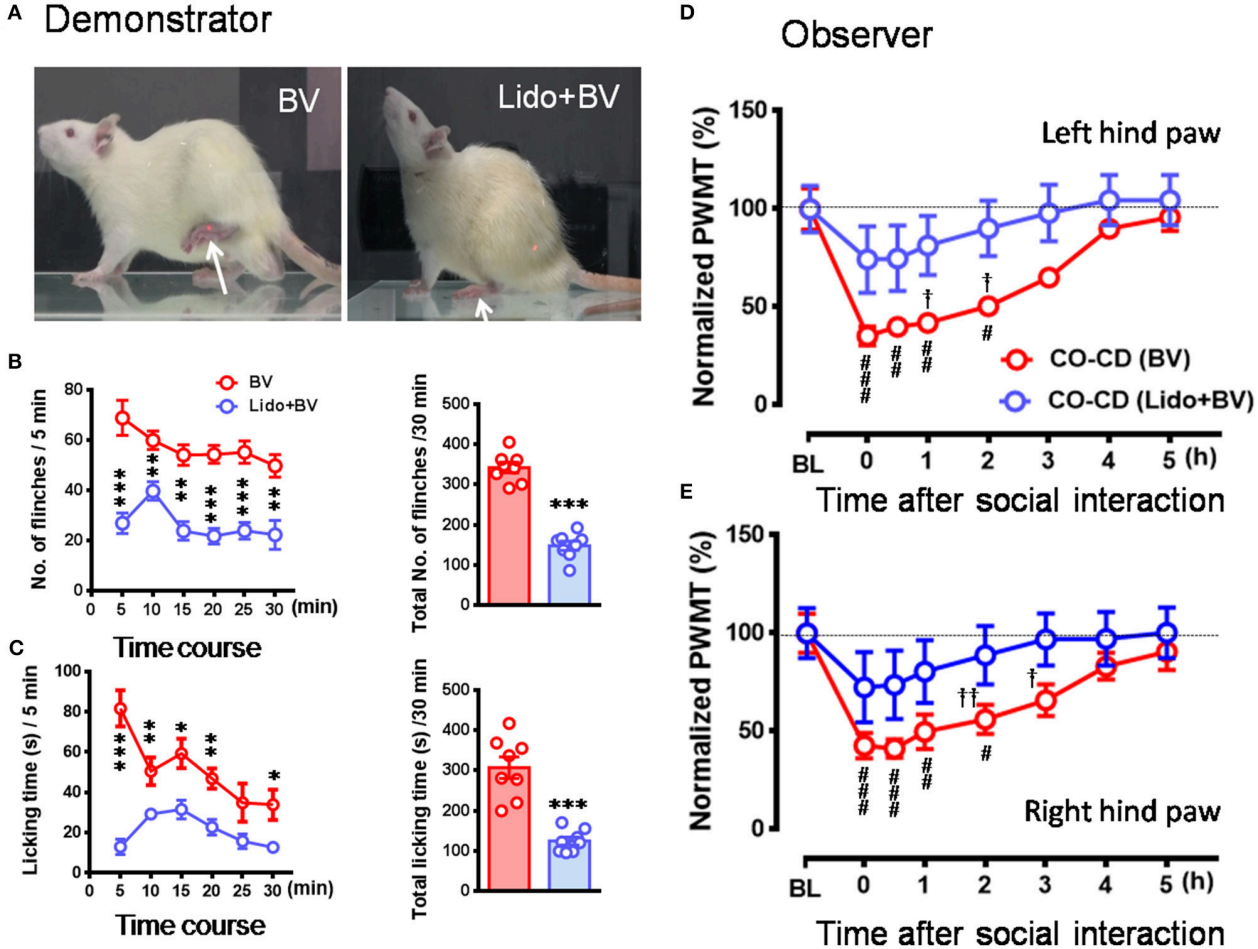
The relieved empathic pain response in the observer after dyadic social interaction with a familiar demonstrator whose pain had been pre-blocked by local analgesia. **(A–C)** Showing analgesic effects of local administration of lidocaine (Lido) on the BV-induced pain-related behaviors (paw flinches and licking) in the demonstrator rats compared with BV only control. **(D,E)** Showing time courses of % changes in PWMT on bilateral hind paws of naïve observer (CO, *n* = 10) after 30 min dyadic social interaction with a familiar demonstrator (CD, *n* = 10) whose pain had been pre-blocked by local analgesia. BL, baseline; BV, bee venom; Lido+BV, local co-administration with lidocaine and BV; CD, cagemate demonstrator; CO, cagemate observer; PWMT, paw withdrawal mechanical threshold. ^*^P < 0.05, ^**^P < 0.01, ^***^ *P* < 0.001 in **(B,C)**, Lido+BV (*n* = 8) vs. BV (*n* = 8) (Mann–Whitney *U*-test and independent *t*-test); #*P* < 0.05, ##*P* < 0.01, ###*P* < 0.001 in **(D,E)**, vs. BL, one way repeat measures ANOVA with Greenhouse-Geisser Correction; ^†^*P* < 0.05, ^††^*P* < 0.01 in **(D,E)**, CO(BV)vs. CO (Lido+BV)with Mann-Whitney U-test. Data are expressed as mean ± SEM.

To see whether pre-blockade of social partner's pain can relieve empathy for pain in the naïve observer rat, CD rats were treated with local co-administration of Lido and BV. As a consequence, while the number of paw flinches and the time that CD rats spent on licking during 30 min priming social interactions were being greatly blocked by local analgesia (Figures 5A–C, ^*^P < 0.05, ^**^P < 0.01, ^***^P < 0.001, Lido + BV [*n* = 8] vs. BV [*n* = 8] for both flinch and licking with Mann–Whitney *U*-test), the EMPH in the CO rats were significantly relieved by shortening the time course and decreasing the amplitude by about 40% [Figures 5D,E, ^†^*P* < 0.05, ^††^*P* < 0.01, CO [Lido+BV] vs. CO [BV] for both paws with Mann-Whitney U-test]. The proportional rate of CO rats showing the EMPH was also decreased from 80% (8/10 for BV only) to 50% (5/10 for Lido+BV) although statistical significance was not reached (2-sided *P* = 0.35, Fisher's two-tailed test).

### Repeated exposures to social partner in pain enhanced EMPH in the naïve observer rat

**Figure 6:**
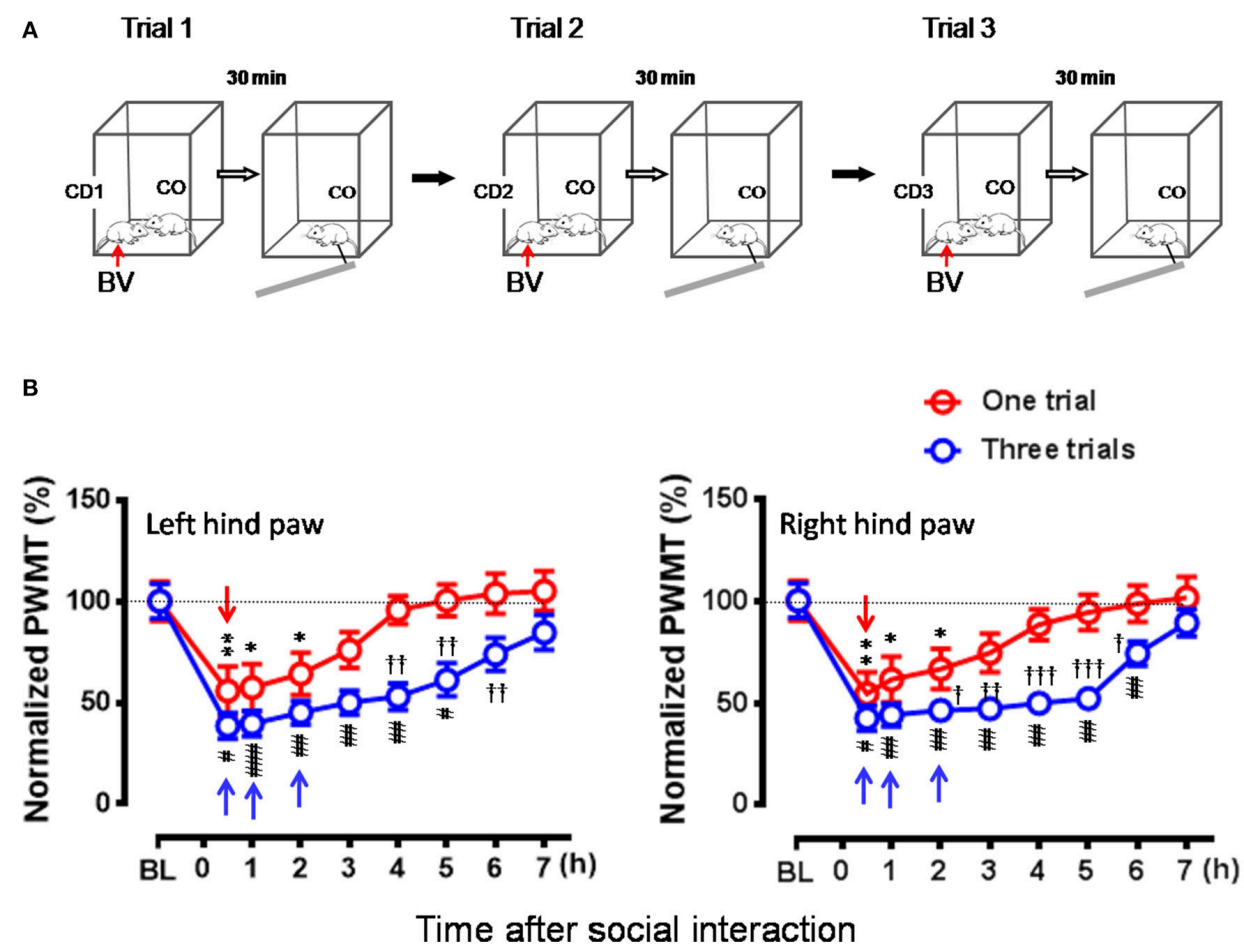
Effects of frequent exposures to a familiar demonstrator in pain on the empathic pain responses in naïve observer. **(A)** Showing an experimental protocol that allowed a naïve observer (CO, *n* = 10) to repeatedly interact with a familiar demonstrator (CD1–3, *n* = 30) in pain successively for three trials (Trials 1–3). In each trial, the dyad interacted socially for 30 min. The values of PWMT in the CO was measured after each trial with 30 min interval. Different CD (CD1–CD3) rat was used for individual trial. **(B)** Showing comparison of the time courses of % changes in PWMT in bilateral hind paws between naïve CO rats who socially exposed to the CD for once (Trial 1) and for three times (Three trials). BL, baseline; BV, bee venom; PWMT, paw withdrawal mechanical threshold. ^*^P < 0.05, ^**^P < 0.01, and #*P* < 0.05, ##*P* < 0.01, ###*P* < 0.001 vs. BL (one way repeat measures ANOVA with Greenhouse-Geisser Correction); ^†^*P* < 0.05, ^††^*P* < 0.01 Three trials vs. One trial with Mann-Whitney U-test. Data are expressed as mean ± SEM. **(A)** Red arrows indicate injection of BV; **(B)** Red downward arrows indicate start of trial 1, while blue upward arrows indicate trial 1 and trial 2 and start of trial 3, respectively.

In this set of experiment, a naïve CO rat allowed to interact with a CD in pain successively for three trials (Figure 6A), while the animals interacting for only one trial served as control. As a result, the time course of the EMPH in CO rats was distinctly elongated from 4 to 7 h until full recovery compared between three trials of exposures to CD rats in pain and one trial (Figure 6B, *P* < 0.001, Three trials vs. one trial with Mann-Whitney U-test). Moreover, the percent decrease in PWMT was also enhanced for the whole time course of observation in general in the group of rats with three trials of exposures compared to one trial control (Figure 6B). Specifically, there was statistically significant difference in % PWMT changes between three trials group and one trial group during 2–6 h after initial social interactions [^†^*P* < 0.05, ^††^*P* < 0.01, ^†††^*P* < 0.001, three Trials vs. one Trial for both paws with Mann-Whitney U-test]. The proportional rate of CO rats showing the EMPH was 80% (8/10) for Trial 1 and 90% (9/10) for both Trial 2 and Trial 3 (2-sided *P* = 1.000, Fisher's two-tailed test).

### Social transfer of mechanical pain hypersensitivity from a rat with chronic pain to a naïve co-housed cagemate

**Figure 7:**
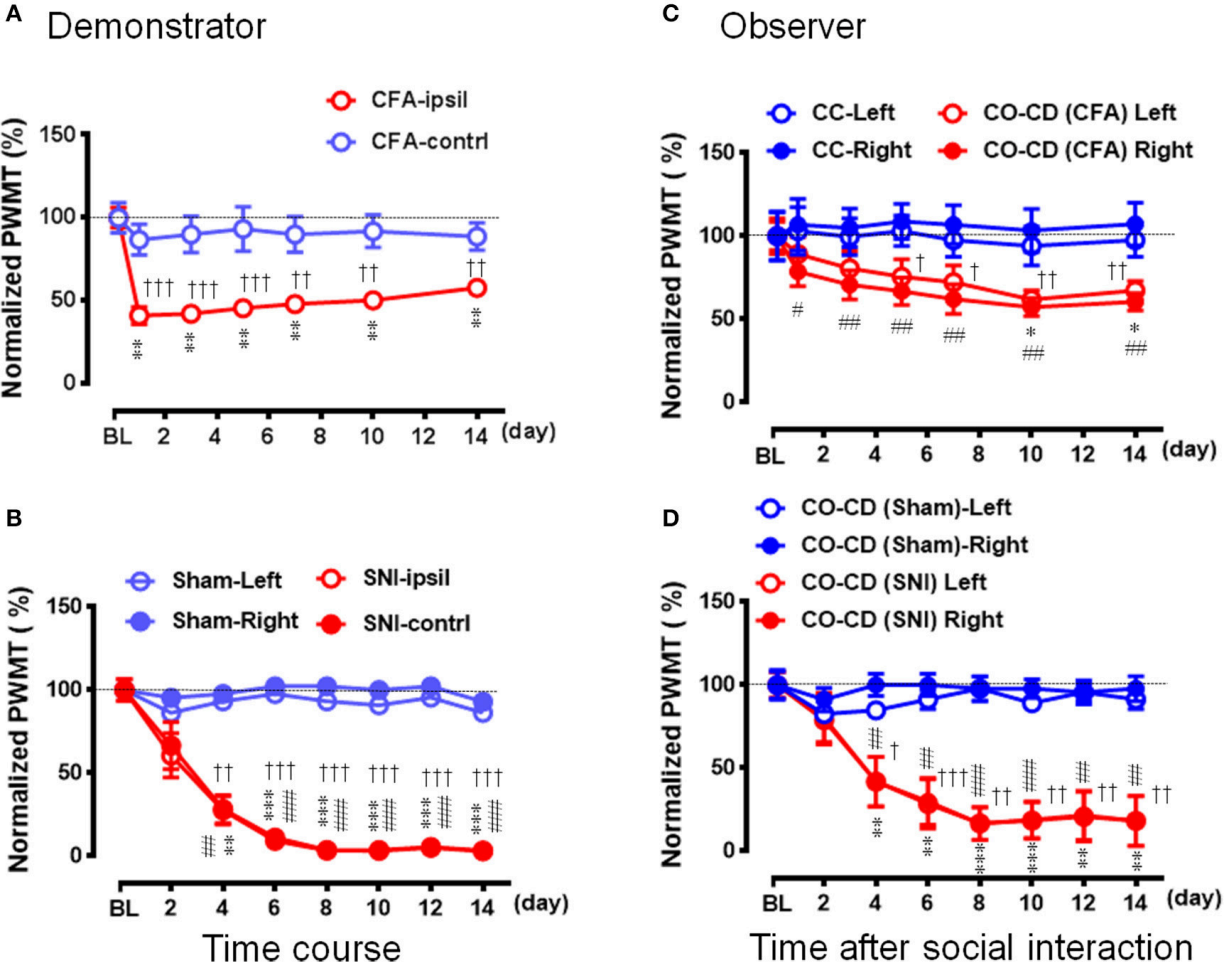
Social transfer of mechanical pain hypersensitivity from a rat with chronic pain to a naïve cagemate while being co-housed in a cage. **(A,B)** Showing the time courses of % changes in PWMT on bilateral hind paws of demonstrators (CD) who suffered from chronic pain caused by peripheral tissue injury (**A**, CFA model, *n* = 10) or peripheral nerve injury (**B**, SNI model, *n* = 9). Rats with sham operation (Sham) served as control for the SNI group. **(C,D)** Showing the time courses of % changes in PWMT on bilateral hind paws of cagemate observers (CO, *n* = 10 for each model) who were being co-housed with a CD rat in pain caused by CFA [CO-CD (CFA)] or SNI [CO-CD (SNI)]. CC (*n* = 6) and CO-CD (Sham) (*n* = 7) were cagemate controls who were being co-housed with naïve or sham rats. BL, baseline; CC-Left/-Right, CO-CD (CFA) Left/Right, CO-CD (Sham) and CO-CD (SNI) Left/Right indicate left or right hind limb of CO rats; CFA, complete Freund's adjuvant; CFA-ipsil/contrl, CFA injection side or contralateral side; SNI, spared nerve injury; SNI-ipsil/contrl, SNI side or contralateral side. ^*^P < 0.05, ^**^P < 0.01, ^***^P < 0.001, and #*P* < 0.05, ##*P* < 0.01, ###*P* < 0.001 vs. BL (one way repeat measures ANOVA with Greenhouse-Geisser Correction). ^†^*P* < 0.05, ^††^*P* < 0.01, ^†††^*P* < 0.001 CFA-ipsil vs. CFA-contral, SNI *vs*. Sham, CO-CD (CFA) vs. CC and CO-CD (SNI) vs. CO (Sham) with Mann–Whitney *U*-test in **(A–D)**. Data are expressed as mean ± SEM.

Because it was shown, in the above experiments, that the CO rats did not develop the EMPH after socially interacting with a CFA-treated CD rat at the beginning 30 min of its pain course, we thus investigated whether pain could be contagious while a CO rat was being co-housed in pairs with an ill CD with chronic pain induced by CFA (Figure 7A) or SNI (Figure 7B) for long time (2 weeks). As shown in Figure 7A, following treatment with CFA, the mechanical pain hypersensitivity was only identified on the injection side of hind paws in the CD rats and it developed quickly by 59% decrease in PWMT on day 1 that was maintained unrecovered at the end of observation (^**^P < 0.01 *vs*. PWMT_baseline_, *n* = 10, One way repeat measures ANOVA with Greenhouse-Geisser Correction). The difference in % change in PWMT between the ipsilateral and the contralateral side in CD rats was statistically significant (^††^*P* < 0.01, ^†††^*P* < 0.001, CFA-ipsil vs. CFA-contrl with Mann-Whitney U-test). While being co-housed with the CFA-treated rats, the bilateral PWMTs in the CO-CD (CFA) rats were significantly decreased on day 1 and remained unrecovered until termination of the observation on day 14 (Figure 7C, ^*^/#*P* < 0.05, ##*P* < 0.01 vs. PWMT_baseline_, *n* = 10, One way repeat measures ANOVA with Greenhouse-Geisser Correction). The % change in PWMT of both hind paws in CO-CD (CFA) rats was significantly different from the co-housed CC rats (Figure 7C, ^†^*P* < 0.05, ^††^*P* < 0.01, CO [CFA] vs. CC for both paws with Mann-Whitney U-test]. The proportional rate of CO-CD (CFA) rats with contagious mechanical pain hypersensitivity was only 30% (3/10) on day 1, but reached 90% (9/10) on day 14 (2 sided *P* = 0.02, day 1 *vs*. day 14 with Fisher's two-tailed test).

Compared with the sham control (*n* = 7), the mechanical pain hypersensitivity was identified on both hind paws in CD rats with SNI and it developed slowly by 40% decrease in PWMT on day 2, by about 70% on day 4 and reached to the plateau level (by 88–97%) between days 6–14 (Figure 7B, ^**^/##*P* < 0.01, ^***^/###*P* < 0.001 vs. PWMT_baseline_, *n* = 9, One way repeat measures ANOVA with Greenhouse-Geisser Correction). The difference in time course % change in PWMT between the Sham and SNI in CD rats was statistically significant (^††^*P* < 0.01, ^†††^*P* < 0.001, SNI vs. Sham, *n* = 9 for both hind paws with Mann-Whitney U-test). While being co-housed in pairs with the SNI-treated rats, the bilateral PWMTs in CO-CD (SNI) rats were also decreased in a parallel manner during the 14 days observation (Figure 7D, ^**^/##*P* < 0.01, ^***^/###*P* < 0.001 vs. PWMT_baseline_, *n* = 9 for both hind paws, One way repeat measures ANOVA with Greenhouse-Geisser Correction). The difference in time course % change in PWMT between the CO-CD (SNI) and CO-CD (Sham) was statistically significant (^†^*P* < 0.05, ^††^*P* < 0.01, ^†††^*P* < 0.001, SNI vs. Sham, *n* = 9 for both hind paws with Mann–Whitney *U*-test). The proportional rate of CO rats with contagious mechanical pain hypersensitivity was about 33% (3/9) on day 2, but reached about 89% (8/9) on day 14 (2 sided *P* = 0.05, day 2 vs. day 14, *n* = 9 with Fisher's two-tailed test).

## Discussion

### Validating the rat model of empathy for pain

In the current study, the rat model of empathy for pain was validated by the following results: (1) The development of empathy for pain in a naïve observer rat is determined not only by the extent of familiarity between the social partners demonstrated previously (Li et al., 2014; Martin et al., 2015; Lü et al., 2017; Lu et al., 2018; for review see Chen, 2018), but also by the expressions of pain-related behaviors in the demonstrators in pain or distress during short period of social interaction. It is likely that, at the first contact, the more displayable or eye-identifiable the SPRBs are in the demonstrators, the much more easily the empathic pain responses can be induced in the naïve observers among familiars. In this meaning, the acute pain models such as the BV test, the formalin test and even the acetic acid writhing test are likely to be more valid for induction of empathy for pain in both rats and mice (Langford et al., 2006; Li et al., 2014; Lü et al., 2017; Lu et al., 2018). However, other pain models such as the CFA test and the late phase of the BV test that have less eye-identifiable SPRBs are not likely to be valid for induction of empathy for pain in rats during short period of social interaction. Empathic mechanical pain hypersensitivity (EMPH) could be seen in 80% of naïve COs following 30 min social interaction with a CD in eye-identifiable pain, however, < 40% was seen in those naïve COs following the same social interaction with a CD in eye-unidentifiable pain. (2) The pre-blockade of SPRBs in the demonstrator rats by local analgesia can significantly relieve the extent of empathic pain responses and decreases the number of animals with empathy for pain in naïve observer rats, further supporting the influencing role of the demonstrator's pain expressions in induction of empathy for pain in rats. Because the empathic pain response of CO can be suppressed by effective analgesia in painful CD, the rat model of empathy for pain is also likely to be useful in screening and/or evaluating the analgesic effects of drugs on emotional component of pain that is socially transferred from familiar conspecific in pain. (3) Repeated exposures to familiar conspecifics in pain may enhance empathic pain responses in both amplitude and time course. Repeated exposures to familiar conspecifics in pain may also increase the proportional rate of CO rats with empathy from 80 to 90%. These results suggest that the neural processes of empathy for pain may be similar to those of learning and memory which can be consolidated by repeated exposures to the visual cue-based environment (Baddeley et al., 2002). (4) Non-displayable or eye-unidentifiable mechanical pain hypersensitivity can also be contagious and transferrable from an ill cagemate with chronic pain to a naïve rat when being co-housed in pairs for at least several days and it was maintained without any recovery until the termination of the observations in the current study (2 weeks in this study). This co-housing contagious pain has also been reported in mice more recently (Baptista-de-Souza et al., 2015). In that mouse study, following being co-housed in pairs with a cagemate in pain caused by the sciatic nerve constriction (another animal model of neuropathic pain) for 14 days, the naïve cagemate mice showed enhanced writhing responses to intraperitoneal (i.p.) injection of acetic acid and anxiety-like (but not depression-like) behaviors, suggesting existence of social transfer or contagion of both pain and pain-related (e.g., anxiety-like) emotions (Baptista-de-Souza et al., 2015). The social contagion or transfer of negative emotions such as anxiety-like and depression-like behaviors have also been demonstrated in normal cagemate mice following 6–8 weeks of co-housing in pairs with a chronically distressed conspecific with pilocarpine-induced temporal lobe epilepsy or chronic depression elicited by chronic foot-shock stress (Yang et al., 2016). The social transfer or contagion of negative emotions between familiar mice has also been shown to result in chronic dysfunctions in social approach, social ability and novel object recognition (Yang et al., 2016). Taken all these results together, it is suggested that the vicariously social contagion or transfer of pain (vicariously felt pain) between familiar rats is dependent upon not only the expressions of pain in social partners but also the time that the dyads spent in social communications. Displayable or eye-identifiable pain expressions of a social partner can cause immediate empathic pain responses in familiar observer rats, leading to reduction of PWMT in bilateral hind paws, however, non-displayable or eye-unidentifiable chronic distress or pain requires longer time for the rat to sense, recognize and share its cagemate's emotional state through more sources of social communications and physical contact. Whether the two types of vicariously felt pain have the same or different underlying mechanisms is not known and requires to be further studied. It has been demonstrated that empathy for pain induced by eye-identifiable SPRBs is mainly dependent upon visual but not olfactory and auditory information (Langford et al., 2006). However, the visual information is not likely to be the only sensory source for development of the long-term co-housing contagious pain hypersensitivity. In one previous report, olfactory cues have also been suggested to be involved in mediation of the transfer of both mechanical and thermal pain hypersensitivity from inflammatory or morphine/alcohol withdrawal painful conspecifics to the bystander mice. In that study, mice did not allow to interact with each other in a socially, physically contactable environment, but instead, they were housed and tested in the same bedding room which inflammatory or morphine/alcohol withdrawal painful conspecifics had been housed before (Smith et al., 2016). Moreover, a period of at least 24 h exposure to the bedding from hyperalgesic mice was shown to be essential for social transfer of pain hypersensitivity to other bystander mice (Smith et al., 2016). Although the olfactory cue-based transfer of pain hypersensitivity may not be empathic response as seen in our and other studies (for review see Chen, 2018), it would be of particular interest to see what is going to happen at the neural circuit level and the molecular/cellular levels in the brain between the two different models because both types of contagious pain involve the mPFC (Li et al., 2014; Smith et al., 2017). Based upon the results of previous and our present studies, we propose that the visual information be more critical for induction of empathy for pain or distress at the first sight or short duration of social communication, while other sensory information such as olfactory, auditory, and somatic sources should also be necessary when salient visual information is lacking. Nonetheless, the exact roles of different sensory inputs in social transfer of pain in general and empathy for pain in particular among social animals and human beings are worth of being further studied.

### Is empathy for pain or distress a highly conserved pro-social behavior?

Do animals and humans have similar affective feelings? It has long been believed that empathy is a unique nature of human beings (for historical reviews see Panksepp, 1998; Preston and de Waal, 2002; de Waal, 2008; de Waal and Preston, 2017; Chen, 2018). However, as early as in the beginning of 1870s, Darwin asserts that “many animals, however, certainly sympathize with each other's distress or danger” (Darwin, 1879; for review also see Chen, 2018). de Waal and his colleagues have provided with a series of long-term observations in apes' reservations that strongly demonstrates existence of empathy and empathic concern in non-human primates (Preston and de Waal, 2002; de Waal, 2008; de Waal and Preston, 2017). Actually, in the late of 1950s Russell Church published the first experimental research work showing emotional reaction of rats to the pain of others in which it was shown that an observer rat stopped getting food via bar pressing while witnessing a conspecific's distress induced by electrical foot shock (Church, 1959). It has also been demonstrated that rats show “altruistic” behavior by pressing bars more frequently to release a distressed conspecific suspended by a hoister while witnessing the visual and auditory signs of discomfort (Rice and Gainer, 1962). In the past decade, the pro-social and empathic like behaviors have been further demonstrated to exist in rodents, showing: (1) development of empathic pain responses (decrease in pain threshold or increase in pain responses) or consolation (allogrooming) in mice (Langford et al., 2006), rats (Li et al., 2014; Lü et al., 2017; Lu et al., 2018) and voles (Burkett et al., 2016) while or after socially interacting with a cagemate in pain or distress caused by pain-producing chemical irritants or electrical foot shock; (2) development of empathic fear responses (increase in freezing) in both rats (Knapska et al., 2006; Chen et al., 2009; Bruchey et al., 2010; Kim et al., 2010; Atsak et al., 2011) and mice (Jeon et al., 2010; Keum et al., 2016) while witnessing or hearing a cagemate in auditory conditioned fear; (3) possession of a learned ability of rats to help release a distressful cagemate from a closed restrainer (Ben-Ami Bartal et al., 2011, 2014); (4) existence of a cooperative capacity of rats to help a social partner in food-seeking (Márquez et al., 2015). The pro-social and empathic like behaviors have also been seen in other social animals such as birds (Watanabe and Ono, 1986; Edgar et al., 2011) and ants (Nowbahari et al., 2009). Collectively, it is likely that empathy for distress (e.g., pain, fear, and even social loss) is a highly conserved pro-social behavior in both mammals and other social living organisms (de Waal and Preston, 2017; Chen, 2018). Although the underlying mechanisms of empathy in general and empathy for distress in particular are less known, some more recent results from both human neuroimaging studies and bio-psychosocial-behavioral paradigm in rodents highly support that empathy for distress is an evolutionary behavior driven by highly conserved biological basis (for reviews see Panksepp and Lahvis, 2011; Gonzalez-Liencres et al., 2013; Panksepp and Panksepp, 2013; Martin et al., 2014; Mogil, 2015; Keum and Shin, 2016; Meyza et al., 2016; Sivaselvachandran et al., 2016; de Waal and Preston, 2017; Chen, 2018).

Consolation behavior has long been believed to be a pro-social and empathic response to other's pain or distress in social animals and human beings (Darwin, 1879; Preston and de Waal, 2002; de Waal, 2008; de Waal and Preston, 2017). More recently, it has been demonstrated that allo-grooming behavior, namely partner-directed grooming toward a stressed familiar conspecific (but not stranger), is ACC-mediated and oxytocin-dependent consolation behavior in prairie vole (Burkett et al., 2016). Allo-grooming behavior has also been seen in rats when socially interacting with a conspecific but it can selectively be enhanced by familiar conspecific in pain (but not stranger) (Lu et al., 2018). In the current study, besides allo-grooming behavior, we also identified allo-licking behavior in CO rats, namely injury site-directed licking toward a familiar conspecific in pain caused by s.c. injection of BV although this behavior was not frequent enough in rats to be quantified separately (see Figure 2 and Supplemental Video 2). Allo-licking behavior was more specific than allo-grooming behavior in terms of empathy and consolation for pain or distress because it could not be identified in the CC during social communication.

It is intriguing to ask whether there is a causal relationship between the consolation behavior and the occurrence of mechanical pain hypersensitivity in CO rats. Although so far direct evidence has not been provided, several lines of indirect evidence might be supportive. First, that familiarity and priming social interaction between the dyads are essential condition to induce mechanical pain hypersensitivity in CO rats supports close link between the two behaviors (Langford et al., 2006; Li et al., 2014; Lü et al., 2017, also see Chen, 2018). Second, as shown in the current study (see Figures 2E, 3D,F), CO rats who developed mechanical pain hypersensitivity had enhanced consolation behavior reflected by spending more time on allo-grooming and allo-licking toward the CD with eye-identifiable spontaneous pain caused by s.c. BV, however, those CO rats who did not develop mechanical pain hypersensitivity had the same level of consolation as the CC when socially interacting with the CD without eye-identifiable spontaneous pain caused by s.c. CFA. Third, as abovementioned, both consolation and EMPH in CO rats have been demonstrated to be oxytocin-dependent and mediated by the mPFC (Burkett et al., 2016; Chen, 2018; and our unpublished data).

### Vicariously felt pain model in animals provides with a novel bio-psychosocial-behavioral paradigm for studying the underlying mechanisms of empathy

Empathy has multiple facets and includes two major domains: affective (emotional) empathy and cognitive empathy that may involve both bottom-up and top-down neural pathways (circuit level) as well as molecular and cellular mechanisms associated with sensory, emotional, cognitive and social components (Bernhardt and Singer, 2012; Decety et al., 2016; de Waal and Preston, 2017; Chen, 2018). Empathy has various definitions since it has appeared in literatures (Leiberg and Anders, 2006). It has recently been defined as “any process that emerges from the fact that observers understand others' states by activating personal, neural and mental representations of that state” (de Waal and Preston, 2017). According to the Russian-doll model for the evolution of empathy, it can also be divided into different levels in a hierarchical order: (1) motor mimicry and emotional contagion; (2) empathic concern and consolation; (3) perspective-taking and targeted helping (de Waal and Preston, 2017). The core of the Russian-doll model is the perception-action mechanism (PAM) that has been proposed to be a theoretical neural model underlying why humans and animals can feel empathy (Preston and de Waal, 2002; de Waal, 2008; de Waal and Preston, 2017). However, on the other hand, the discovery of mirror cells in the monkey's brain has also been thought to provide with another neural basis for empathy (Zaki et al., 2009; Rizzolatti and Sinigaglia, 2016). Debate and controversy still exists between the PAM (de Waal and Preston, 2017; Preston and de Waal, 2017) and the mirror system (Rizzolatti and Caruana, 2017) in terms of theoretical considerations about representations of empathy. Recently, Zaki provided with a motivated model for empathy in which he proposes that empathic response be determined by personal motivations that can be modulated by social approach (capitalization, affiliation, and desirability) and social avoidance (pain, cost, and interference) through situation selection, attention, and appraisal when he/she exposes to and perceives the target cue (Zaki, 2014). However, most of the existing theoretical ideas about empathy are anthropocentric. From the point of evolutionary view, empathy can be simply referred to as an evolutionary behavior of social animals and humans associated with prosocial reciprocity, altruism, and morality produced by the ability and capacity to feel, recognize, understand, and/or share the emotional states of others (Chen, 2018). As such, the behavioral processes of empathic responses are likely to be associated with individual's sensory, emotional, cognitive and social experiences that are critical for both family/in-group survival (maternal/kinship care, share, trust, cooperation, conformity) and out-group competition (defense and aggression) in social animals. These behavioral processes associated with social approach and social stress are likely to be driven or governed by both the neural representations (neural circuits, synaptic plasticity, cellular and molecular events, etc.) in the central nervous system and the neuroendocrine regulation in the peripheral organs through circulating hormones (Neumann et al., 2000; Heinrichs et al., 2003; Ulrich-Lai and Herman, 2009; Lukas et al., 2011; Li et al., 2014; Lü et al., 2017). Among the multiple faces of empathy, as described above, empathy for distress (vicariously felt pain and fear) is a set of highly conserved pro-social behaviors that is fundamental to and critical for family/in-group survival across different species. Vicariously felt pain (or fear) model in animals may provide with a novel bio-psychosocial-behavioral (BPSB) paradigm for studying the underlying mechanisms of empathy. Although so far less is known about the underlying mechanisms of empathy, some published data obtained from animal models highly support the usefulness and effectiveness of the BPSB paradigm. First, vicariously felt pain (or fear) seen in rodents is likely to be parochial and familiarity-dependent (Darwin, 1879; Gonzalez-Liencres et al., 2013; Li et al., 2014; Martin et al., 2014; Mogil, 2015; Lü et al., 2017; Lu et al., 2018; Chen, 2018) that is largely consistent with the phenomenon seen as empathy for distress (pain, fear and even social loss) in humans (Singer et al., 2004; Lamm et al., 2011; Iannetti et al., 2013; Eisenberger, 2015; De Dreu and Kret, 2016; Han, 2018). Second, vicariously felt pain (or fear) seen as empathy for distress among familiar rodents has been demonstrated to be mediated by the mPFC (Jeon et al., 2010; Li et al., 2014; Burkett et al., 2016) where has also been demonstrated to be one of the major brain regions activated by empathy for distress (pain and social loss) in humans (Singer et al., 2004; Lamm et al., 2011; Iannetti et al., 2013; Eisenberger, 2015). Third, vicariously felt pain (or fear) seen as empathy for distress among familiar rodents has been shown to be facilitated by both hypothalamic-neurohypophysial (oxytocin) system (Neumann et al., 2000; Heinrichs et al., 2003; Lukas et al., 2011; Burkett et al., 2016) and the SAM system (LC-NE system) (Lü et al., 2017) which have been demonstrated to be essential for induction of empathy and pro-social behaviors in humans (Insel and Young, 2000, 2001; Donaldson and Young, 2008; Heinrichs and Domes, 2008; Lee et al., 2009; Terbeck et al., 2016). Fourth, vicariously felt pain (or fear) seen as empathy for distress has been demonstrated to be prevented by activation of the HPA axis in both humans and animals due to social stress (Neumann et al., 2000; Heinrichs et al., 2003; Lukas et al., 2011; Martin et al., 2015; Lü et al., 2017). Taken together, vicariously felt pain transferred from social interaction with a familiar conspecific in pain is a valid rodent model for studying the underlying neural mechanisms of empathy for distress at both neural circuit level and molecular/cellular levels.

## Conclusions

The vicariously social contagion or transfer of pain between familiar rats is dependent upon not only the expressions of pain in social partners but also the time that the dyads spent in social communications. The rat model of empathy for pain is a highly stable, reproducible and valid model for studying the neural mechanisms of empathy in lower animals.

## Ethics statement

This study was carried out in accordance with the recommendations of the ARRIVE guidelines (Kilkenny et al., 2010), the U.K. Animals (Scientific Procedures) Act 1986 and associated guidelines, the EU Directive 2010/63/EU for animal experiments, the National Institutes of Health guide for the care and use of laboratory animals (NIH Publications No. 8023, revised 1978), and The ethical guidelines for investigations of experimental pain in conscious animals of the International Association for the Study of Pain were also critically followed (Zimmermann, 1983).

## Author contributions

JC and C-LL conceived the study. C-LL, YYu, TH, X-LW, and YYa performed the experiments and carried out the data analysis, R-RW, K-WG, RD, W-JL, NW, YW, and Y-QY were involved in some experiments and data analysis. JC and C-LL drafted the manuscript.

### Conflict of interest statement

The authors declare that the research was conducted in the absence of any commercial or financial relationships that could be construed as a potential conflict of interest.

## Supplementary material

Supplemental Video 1 Different expressions of pain in demonstrator rats prepared with four animal models of pain: the bee venom test, the formalin test, the complete Freund's adjuvant model and the spared nerve injury model.

Supplemental Video 2 Priming dyadic social interactions between a naïve cagemate observer and a cagemate demonstrator in pain.
